# Surface Modification of Polycarbonate by an Atmospheric Pressure Argon Microwave Plasma Sheet

**DOI:** 10.3390/ma12152418

**Published:** 2019-07-29

**Authors:** Dariusz Czylkowski, Bartosz Hrycak, Andrzej Sikora, Magdalena Moczała-Dusanowska, Mirosław Dors, Mariusz Jasiński

**Affiliations:** 1 Institute of Fluid Flow Machinery, Polish Academy of Sciences, Fiszera 14, 80-231 Gdańsk, Poland; 2 Electrotechnical Institute, Division of Electrotechnology and Materials Science, M. Skłodowskiej-Curie 55/61, 50-369 Wrocław, Poland

**Keywords:** surface modification, polycarbonate, microwave plasma, atmospheric pressure plasma

## Abstract

The specific properties of an atmospheric pressure plasma make it an attractive tool for the surface treatment of various materials. With this in mind, this paper presents the results of experimental investigations of a polycarbonate (PC) material surface modification using this new type of argon microwave (2.45 GHz) plasma source. The uniqueness of the new plasma source lies in the shape of the generated plasma—in contrast to other microwave plasma sources, which usually provide a plasma in the form of a flame or column, the new ones provides a plasma in the shape of a regular plasma sheet. The influence of the absorbed microwave power and the number of scans on the changes of the wettability and morphological and mechanical properties of the plasma-treated PC samples was investigated. The mechanical properties and changes in roughness of the samples were measured by the use of atomic force microscopy (AFM). The wettability of the plasma-modified samples was tested by measuring the water contact angle. In order to confirm the plasma effect, each of the above-mentioned measurements was performed before and after plasma treatment. All experimental tests were performed with an argon of flow rate up to 20 L/min and the absorbed microwave power ranged from 300 to 850 W. The results prove the capability of the new atmospheric pressure plasma type in modifying the morphological and mechanical properties of PC surfaces for industrial applications.

## 1. Introduction

Polycarbonate plastic (PC) is characterised by its specific features, such as transparency, high toughness, high electrical insulation, high temperature resistance, and ease of machining and fabricating [1,2,3,4,5,6]. Due to this, PC material is widely used in optical applications (plastic lenses in eyewear), in food contact materials (water, milk and beverage bottles) [7], in medical devices (high-pressure syringes, surgical instruments, neonatal incubators) [8], and in digital optical data storage discs (CD, DVD, Blu-ray), to list only a few. However, like all plastic materials, PC is characterised by low surface free energy, resulting in poor wetting and adhesion properties [6,9,10]. Commercial use of PC materials most often requires printing on the surfaces of PC-made objects. Thus, to improve the wetting and adhesion properties, the activation of PC surfaces is necessary to improve the bonding of the inks used for printing. Except for old means of mechanical surface activation, there are chemical and physicochemical methods of plastic material surface activation [11]. The characterisation of the selected above-mentioned activation methods can be found in Reference [12] for a high temperature gas flame-based method, in Reference [11] for a chemically active layer of primer-based method, in Reference [13] for an ultraviolet (UV) light-based method, in Reference [14] for an ozone-based method, and in Reference [15] for an atom beam-based method, among others. One of the treatment and modification method of various materials surfaces (thus also activation of their surfaces) is the use of plasma. In comparison to other surface treatments and modification methods, the plasma-based method exhibits some advantages. First of all is that plasma can change the treated surface properties without changing the bulk properties. In contrast to chemical-based surface activation methods, plasma surface modification does not use hazardous chemicals (solvents, acids, alkalies) and does not require a large amount of water. In comparison to flame-based methods, plasma surface modification does not involve a high-temperature flame of explosive gas, such as propane or acetylene. Thus, from this point of view, plasma surface modification is an environmentally friend process. The results of experimental investigations of the modification of different plastic material surfaces by plasma has been reported, for example, in References [16,17,18,19,20,21,22,23,24,25,26]. In References [27,28,29,30], recently published results on the plasma-induced surface functionalization of polymers can be found. These results relate to surface modifications by plasma of such materials as polyethylene (PE), polypropylene (PP), polytetrafluoroethylene (PTFE), polyvinyl chloride (PVC), polyester (PS), and others. Plasma-based surface activation has also been applied for polycarbonate material [31,32,33,34,35,36,37,38,39,40,41,42,43]. As was mentioned above, plasma has been used for surface modifications of various kinds of plastic materials. At the same time, different types of plasmas have been used in terms of the kind of plasma-forming gas, the pressure, and the method of plasma generation. For instance, considering the methods of plasma generation, DC plasma [20,23,36,37,38], DBD plasma [18,35,40], corona discharge [42,43], RF plasma [17,19,20,22,31,32,34,39], and microwave plasma [25,33] have been used for the modification of different plastic materials.

Among all of the plasma sources for surface activation, the use of atmospheric pressure microwave plasma sources seems to be more desirable. This is because of the undoubted advantages of microwave atmospheric pressure plasma sources. Operation at atmospheric pressure, in contrast to sources using a vacuum apparatus, simplifies the surface activation method and reduces the operational costs. Using a microwave frequency of 2.45 GHz allows cheap commercial magnetrons (such as those installed in microwave ovens) and standard microwave components to be used. A properly designed microwave plasma source can achieve nearly one hundred percent energy transfer efficiency from the microwave generator into the plasma. Another merit is the possibility of electrodeless operation, allowing a plasma of high purity to be generated. As a consequence of this, we designed and built a novel atmospheric pressure microwave plasma source for the treatment of material surfaces that meets all of these requirements. However, more importantly, the major advantage of this novel atmospheric pressure microwave plasma source is the unique shape of the generated plasma, having the form of a regular sheet. Such a shape of plasma is convenient for the treatment of large surfaces and surfaces of different geometries, eliminating time- and cost-consuming multiple scans of the modified surface to cover its full area. This simple, low cost, and easy to operate novel microwave plasma source can be scaled up relatively easily to an industrial production line for material surface modification. The characteristics of the novel microwave plasma source and properties of the plasma sheet in terms of visualisation of the plasma sheet, optical emission spectroscopy, simulation of the electric field distributions, and numerical modelling of the plasma-forming gas flow are presented in References [44,45,46].

The goal of this work is to present experimental results of the surface modification of the polycarbonate material by an atmospheric pressure argon microwave plasma sheet. The roles analysed included the microwave power supply to the plasma sheet and the number of scans on the mechanical properties, sample roughness changes and the wettability of the plasma-treated polycarbonate sample.

## 2. Materials and Methods

### 2.1. Materials

Commercial samples of Makrolon UV polycarbonate material, sized 300 mm × 50 mm and with a thickness of 5 mm, were supplied by Covestro (Pittsburgh, PA, USA). The pieces sized 50 mm × 50 mm, necessary to perform the measurements, were cut before the experiment. The samples’ wettability was determined from measured contact angles of distilled water using a home-made device. Argon (Air Liquide, Kraków, Poland) of purity greater than or equal to 99.998 vol % was used as the plasma-forming gas.

### 2.2. Investigation Methodology

**Figure 1:**
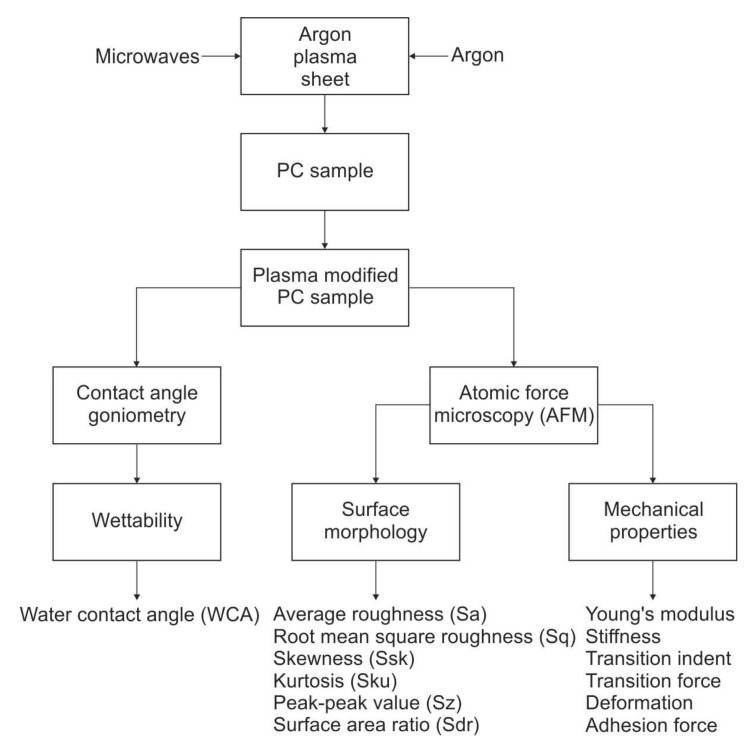
Diagram of the investigation methodology.

An overall diagram of the investigation methodology of the polycarbonate material modification by an atmospheric pressure microwave plasma is presented in Figure 1. A microwave-excited (2.45 GHz) plasma generated in argon at atmospheric pressure was used. Due to the energy transfer from the electromagnetic field of the microwaves into the plasma forming gas (argon), the plasma is generated and sustained inside the flat quartz box and, because of the gas flow, the plasma sheet protrudes outside of the box, permitting the processing of the PC samples’ surfaces. As a result of this, plasma-modified PC samples are obtained. The changes in the wettability of the plasma-modified PC samples’ surfaces were characterised by the water contact angle, measured with the use of standard contact angle goniometry, while the changes in the PC samples’ surface morphology and mechanical properties were investigated by atomic force microscopy (AFM). This diagnostic tool was successfully utilised in the evaluation of submicron changes of the surfaces’ properties after exposure to the plasma [47,48,49,50,51,52]. A detailed description of the PC sample surface modification by an argon microwave plasma, the contact angle goniometry, and atomic force microscopy is provided later in the text.

### 2.3. Polycarbonate Material Surface Modification by an Argon Microwave Plasma

**Figure 2:**
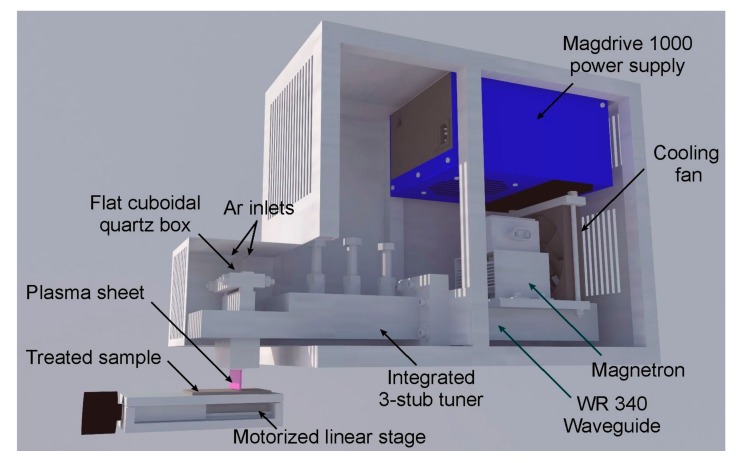
Schematic representation of the novel microwave plasma source for surface modification of polycarbonate materials.

A novel microwave (2.45 GHz) plasma source (MPS) using atmospheric pressure argon [44,45,46] was used for the surface modification of polycarbonate materials. In contrast to the other MPSs operating at atmospheric pressure, which deliver plasmas in the form of cylindrical plasma columns sustained inside a dielectric tube [53,54] or flames protruding outside of the dielectric tube [55,56], or a flame generated at the tip of a nozzle [57,58], the MPS used in the presented paper generates a very conveniently shaped plasma sheet. This type of MPS may be based on a rectangular waveguide or on the strip-line structure [46]. In this experiment, we used a set-up based on a standard WR 340 rectangular waveguide (Figure 2). An argon plasma was generated inside a flat quartz box and protruded out of the box. The flat quartz box was inserted into the waveguide through the rectangular slits in the section of reduced-height of the MPS. The working gas (argon) was introduced into the quartz box by two opposite ducts placed in its upper part. The MPS had an integrated 3-stub tuner to match the impedance. Microwaves were generated by a standard magnetron (CEFEMO 2M240H, Tokyo, Japan), commonly used in kitchenette microwave ovens. The magnetron was powered by a Dipolar MagDrive (Skellefteå, Sweden) power supply. The microwave power absorbed by the plasma was up to 850 W and the argon flow rate up to 20 L/min. In these conditions, the plasma sheet outside the box was about 50 mm in width, 10 mm in length, and 1 mm thick. As it was presented in Reference [25], for the microwave absorbed power of 850 W and the argon flow rate of 20 L/min, the temperature of OH radicals (assumed to be close to the gas temperature) determined using optical emission spectroscopy was about 1100 K. It was also found out that the rotational temperature of OH radicals increases with the increase of the absorbed microwave power and decreases with increasing the argon flow rate [25,44]. The treated polycarbonate samples were placed on a motorised (with stepper motor) linear stage. Thanks to this, linear movement of the PC sample against the plasma sheet was possible. The linear stage was placed 20 mm below the open side of the quartz box. The linear stage moved the PC samples so that the plasma was scanning its area from side to side. The sample transfer speed under the plasma sheet was 10 mm/s.

### 2.4. Contact Angle Goniometry

The surface wettability of the microwave plasma modified PC samples was evaluated from the water contact angle, measured by standard goniometry. For this purpose, a home-made measurement set-up was used. It consisted of a water drop dosing system with a microcontroller, a light source, a microscope with a CCD camera (Opticon, Wrocław, Poland), and a computer with specialised home-made software. The water contact angle was measured immediately after the PC sample plasma treatment; thus, the ageing effect can be neglected. In addition, the test spots provided the necessary conditions to avoid the presence of morphological defects that could affect the results of the measurements. The measurement was carried out with distilled water and in ambient air at room temperature (about 24 °C) and at a relative humidity of 35%. A drop of distilled water was placed dropwise onto the PC sample surface and two images of both sides of the water drop were simultaneously acquired. In this way the obtained images were processed using specialised computer software. To minimise statistical error and assure a certain level of outcome reliability for each PC sample, measurement series of at least 5 drops were conducted.

### 2.5. Atomic Force Microscopy (AFM)

Atomic force microscopy (AFM) was used to investigate the changes in surface morphology, as well as to determine the changes in mechanical properties of the plasma-modified PC samples. In the herein reported experimental investigations, a DI-3000 atomic force microscope (Digital Instruments, Santa Barbara, CA, USA) was used. The DI-3000 AFM provides a maximum scanning range of 100 µm × 100 µm, by a variety of high-resolution surface imaging modes.

To perform roughness changes of the plasma-modified PC samples, the tapping mode [59] was applied. In this case a PointProbe (Nanosensors, Neuchâtel, Switzerland) with nominal tip radius of r_tip_ *=* 10 nm, spring constant of *k =* 43–68 N m^−1^, and a resonance frequency range of *f*_res_ = 306–353 kHz was used. To prevent undesirable effects due to imperfections of the samples’ surfaces, the scanning area was chosen to be 3 µm × 3 µm, which also assures that the desired resolution is achieved, which allows features at the nanometre scale to be observed. In order to provide statistically relevant data, at least 10 scans for each sample were acquired. The obtained data was processed and analysed using the SPIP (Scanning Probe Image Processor) software (ver. 5.1.7, Image Metrology, Lyngby, Denmark) [60]. For each image, the following roughness parameters were calculated: Average roughness (Sa), root mean square roughness (Sq), skewness (Ssk), kurtosis (Sku), peak-peak value (Sz), and surface area ratio (Sdr). As Ssk and Sku parameters are not provided commonly in the literature and, in our opinion, are important in terms of the analysis of changes of morphological parameters of the surface, the meaning of those variables is as given below:

Sku—the peak’s width of height distribution providing quantitative information concerning dispersion level of the height values,

Ssk—the level of asymmetry of the height’s values peak. It provides the information if dominating structures are the hills or the pores.

For each of these parameters, the first quartile, median and third quartile were calculated.

To investigate changes in the mechanical properties of the plasma-modified PC samples, the force spectroscopy mode [61] was used. For that purpose, a CSG30 probe (NT-MDT Spectrum Instruments Ltd., Zelenograd, Russia) with a nominal spring constant of *k_nom_* = 0.6 N m^−1^ and resonance frequency of *f*_res_ = 48 kHz was applied. However, to determine the real spring constant of the probe, the thermal noise method described in Reference [62] was applied. To avoid the influence of PC sample surface inhomogeneity, and to deliver representative data, the measurements were carried out in a few spots on each PC sample. The obtained data was processed and analysed in order to obtain statistical information about the average value, first quartile, median, and third quartile of such mechanical parameters as Young’s modulus, stiffness, transition indent, transition force, deformation, and adhesion force.

Similarly, as in the case of contact angle goniometry, the atomic force microscopy measurements were performed in ambient air at room temperature of about 24 °C and at a relative humidity of 35%.

## 3. Results and Discussion

**Figure 3:**
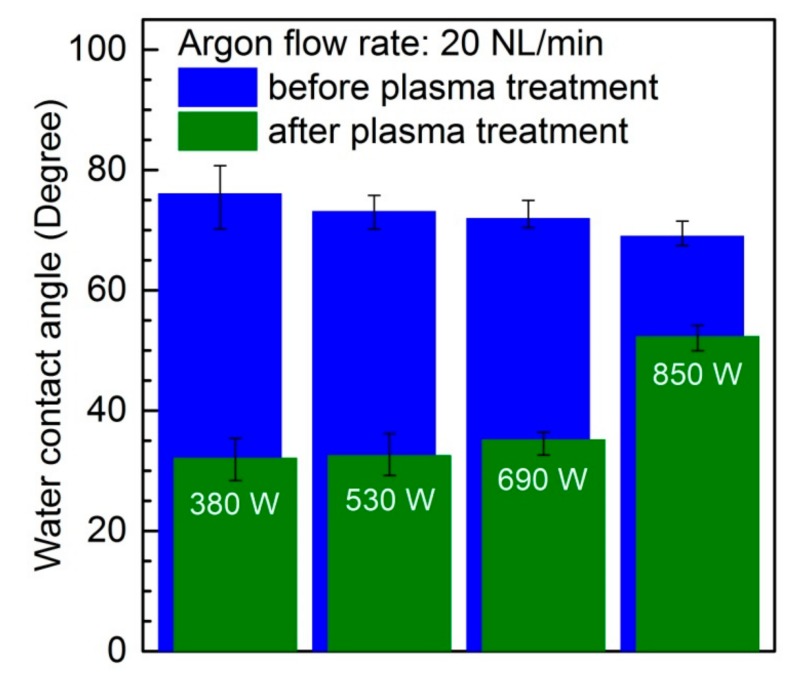
Effect of the microwave power on the water contact angle. Argon flow rate 20 NL/min.

The effect of the microwave power on the water contact angle at an argon flow rate of 20 NL/min is shown in Figure 3. The results correspond to an absorbed microwave power of 380 W, 530 W, 690 W, and 850 W. For each absorbed microwave power, the water contact angle before plasma treatment is given. All of the presented results relate to a single scan (number of scans equal to 1) of the PC sample surface by an argon plasma sheet. To prevent the ageing effect, the water contact angle was measured directly after the plasma treatment. As expected, as it can be seen from the figure, for all of the tested values of absorbed microwave power, the water contact angle decreased after plasma treatment. Depending on the level of absorbed microwave power, the water contact angle dropped from 1.3 times to 2.4 times. The highest change in the water contact angle (2.4 times) was recorded for the lowest absorbed microwave power (380 W). In this case, the water contact angle changes from about 76° for a plasma-untreated PC sample to about 32° if the PC sample was treated with the argon plasma one time. The lowest change in the water contact angle (1.3 times) corresponds to the highest absorbed microwave power (850 W). In the case of absorbed microwave power of 850 W, the plasma treatment led to a decrease of the water contact angle from about 70° to about 52°. As it was mentioned above, the presented results on changes in the water contact angle relate to a single scan of PC sample with the argon plasma sheet. The results of our experimental study with microwave plasma treatment of different polymeric materials show that the effect of decreasing the water contact angle by the second scan is not as significant as after previous scan. In order to exclude the time-consuming multiple scanning, the more appropriate way to further improve the water contact angle is to increase the plasma treatment time. As it can be found in Reference [32] in the case of DC air low pressure plasma treatment of PC samples, in Reference [63] in the case of RF argon low pressure plasma treatment of PC samples, in Reference [21] in the case of DC argon low pressure plasma treatment of PTFE samples, in Reference [36] in the case of DBD air atmospheric pressure plasma treatment of PC samples, and in Reference [18] in the case of AC argon atmospheric pressure plasma treatment of PET samples, the decrease in water contact angle is a plasma treatment time-dependent effect. Thus, it can be expected that by increasing the microwave plasma sheet treatment time, the water contact angle can be further improved.

In Table 1, the comparison of the water contact angle for different plasma-based methods of surface modification of polymeric materials is presented. Different plasma-based methods in the meaning of plasma source type, kind of plasma forming gas, and pressure are considered. Additionally, Table 1 shows the results of the water contact angle not only for polycarbonate material, but also for polytetrafluoroethylene, polyethylene, polypropylene, cyclic olefin copolymer, and cyclic olefinpolymer. The results of the present studies are presented in the last line of the table. With respect to polycarbonate material surface modification, the method presented in this paper involving the argon microwave plasma sheet is comparable with other methods, as presented in Table 1.

It should be mentioned here that, as was reported in e.g., [25,40,42,64,65], the water contact angle after plasma treatment of polymeric materials is not constant, but changes depending on the sample store time. However, as was highlighted in Reference [25] for polyethylene surfaces and in Reference [42] for polycarbonate surfaces, even after 8 days and after two weeks, respectively, the water contact angle did not return fully to the water contact angle of a pristine sample (a sample before the plasma treatment).

Additionally, it should be noted here that, although the changes in the water contact angle of the plasma-treated PC surface sample confirms changes in sample surface morphology, the water contact angle measurements do not deliver as much insight into the morphology changes as atomic force microscopy delivers. Therefore, quantitative data is required.

**Figure 4:**
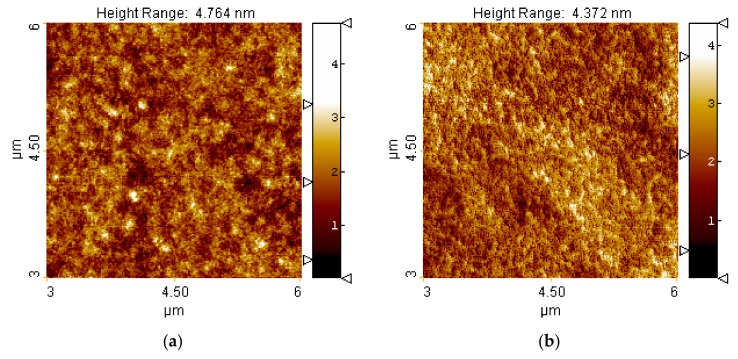
Examples of AFM acquired images of the PC surface: **(a)** Untreated and **(b)** treated using 620 W of microwave power.

Figure 4 shows the results of AFM imaging of the surface of untreated and treated samples. However, the differences can be noticed in the set of quantitative parameters allowed to compare the outcome for wide range of experimental parameters.

**Figure 5:**
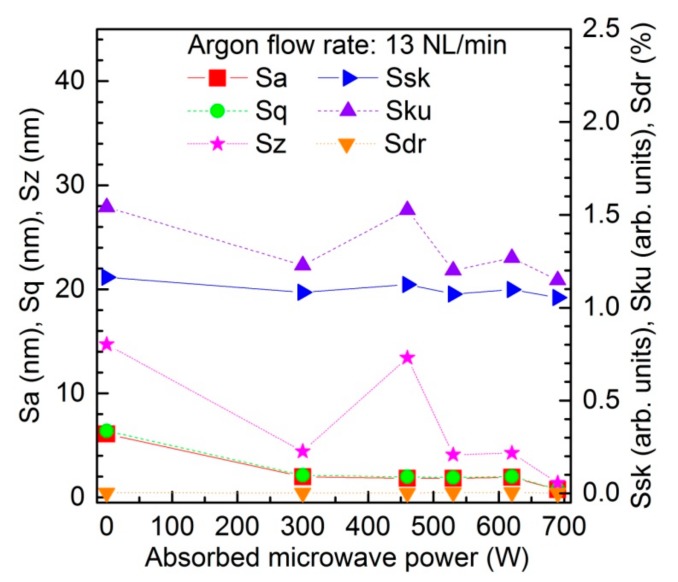
Effect of the microwave power on the roughness parameters (Sa, Sq, Sz, Ssk, Sku, Sdr) of the polycarbonate sample. Argon flow rate 13 NL/min.

Figure 5 illustrates the effect of the microwave power on the roughness parameters of the PC sample. The results corresponded to an absorbed microwave power of 300–690 W and an argon flow rate of 13 NL/min. In the figure, such roughness parameters as average roughness (Sa), root mean square roughness (Sq), peak-peak value (Sz), skewness (Ssk), kurtosis (Sku), and surface area ratio (Sdr) are taken into account. For all of the above-listed parameters, their values for the plasma-unmodified PC sample are given (depicted by 0 W of absorbed microwave power). As can be seen, Figure 4 shows the different changes in the individual surface morphology parameters after plasma treatment with the absorbed microwave power. While the main roughness parameters (Sa, Sq, Sz) decrease with the energy increase, one can also observe the Ssk decrease, indicating the more significant role of the holes in the morphological properties, as well as the Sku decrease, providing the information about the smoothing the height distribution curve. The Sdr parameter also decreases while the energy of the process increases, revealing reduction of the active surface. Yet, the results acquired at 460 W indicate that one may obtain the opposite results, as the larger energies may rapidly disturb the control of the process and lead to an unwanted outcome. Therefore, the alternative approach instead of the high power of the unit application, was the multi-pass of the sample.

**Figure 6:**
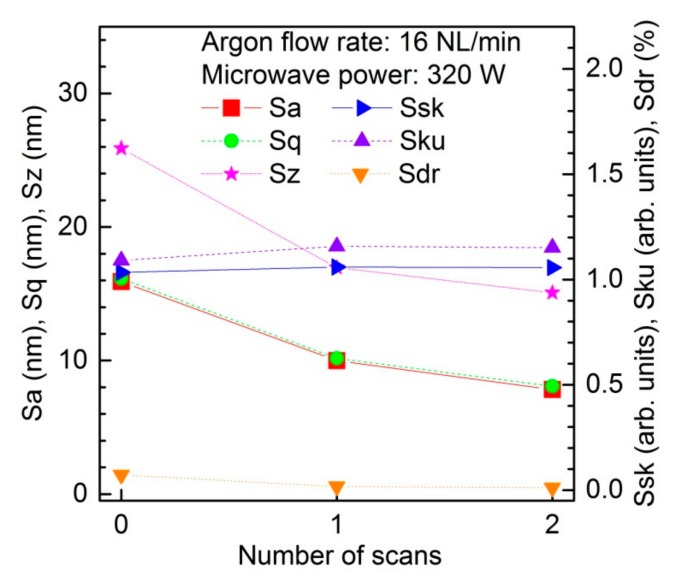
Dependence of the roughness parameters (Sa, Sq, Sz, Ssk, Sku, Sdr) of the polycarbonate sample on the number of plasma scans. Argon flow rate 16 NL/min. Microwave power 320 W.

The dependence of the roughness parameters (Sa, Sq, Sz, Ssk, Sku, Sdr) of the PC sample on the number of plasma scans is shown in Figure 6. The measurements were carried out at an absorbed microwave power of 320 W and at an argon flow rate of 16 NL/min. Depending on the variable being tested, a different number of scans (0–2) with constant speed was performed. In the figure, the results corresponding to the number of scans equal to 0 indicate the results for the plasma-unmodified PC sample. The roughness parameters (Sa, Sq, Sz) decrease along with the following scans. In addition, the Sdr parameter reveals the reduction of the active surface. Additionally, the increase of the Ssk parameter reveals the less significant contribution of the holes in the morphology. Finally, the Sku parameter shows a narrower distribution of the height values, so one can conclude that the process allowed improvement of the surface in terms of morphology, by reduction of the roughness and the presence of holes in particular, while the wettability was improved. Therefore, one can clearly see that the plasma scans changed the surface energy and the effect is exposure-time-dependant. Having the data from both, energy and repetition experiments, one can conclude that in order to improve the surface’s quality by reducing the roughness, one has to consider several repetitions of the low energy procedure than a single pass with large energy. A thermal effect, energy correlated, causing the morphological degradation is very likely involved in such a phenomenon. It should be emphasized that the applying of further plasma processing sessions may cause a change in the morphological parameters change direction. Such non-monotonic appearance was observed in other investigations based on light-polymer interaction [47,48,50,62].

**Figure 7:**
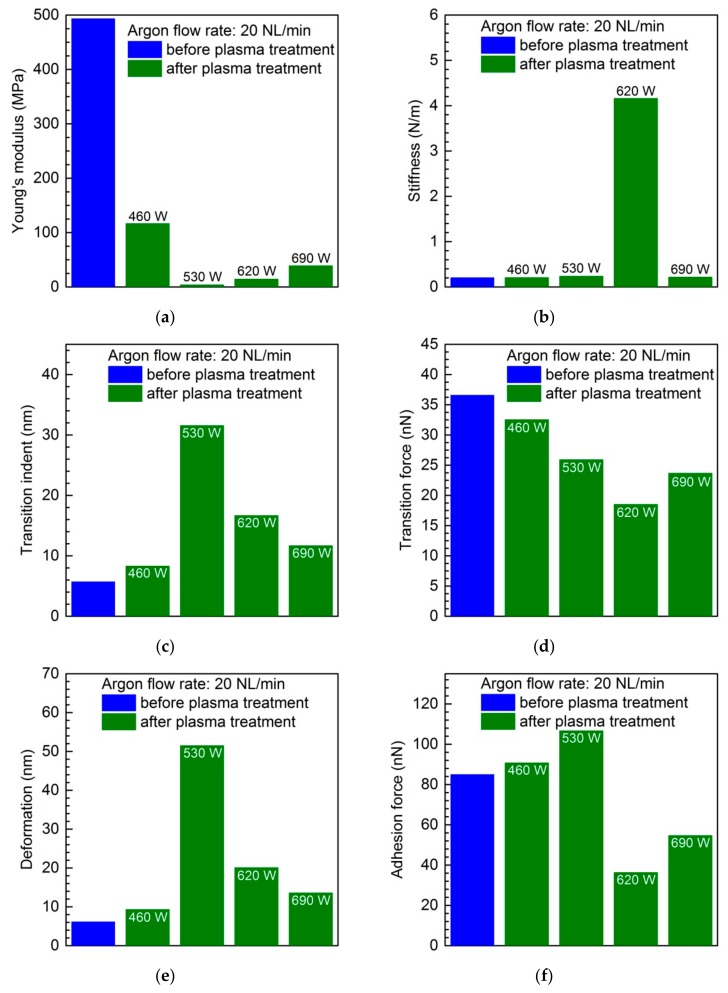
Dependence of the polycarbonate surface mechanical properties parameters: (**a**) Young’s modulus; (**b**) stiffness; (**c**) transition indent; (**d**) transition force; (**e**) deformation; and (**f**) adhesion force on the microwave power. Argon flow rate NL/min.

The dependencies of the polycarbonate surface’s mechanical properties parameters, including Young’s modulus, stiffness, transition indent, transition force, deformation and adhesion force, on the microwave power are shown in Figure 7. The argon flow rate entering the microwave plasma source was set at 20 NL/min, with the absorbed microwave power being varied between 460 W and 690 W. Figure 7a–f show the results for Young’s modulus, stiffness, transition indent, transition force, deformation, and adhesion force, respectively. Similar to the previous results presented in Figure 3, Figure 4, Figure 5 and Figure 6, and also in this case, the proper values for the sample not subjected to plasma modification are given. The results in Figure 7 clearly indicate changes of the above-mentioned parameters caused by the treating of the PC samples by the microwave argon plasma. They are mostly nonlinear and irregular and some of these changes are significant. From Figure 7a, a noticeable decrease of the Young’s modulus at any absorbed microwave level can be observed. In contrast, Figure 7b shows that at absorbed microwave power of 460 W, 530 W, and 690 W, no significant changes in the stiffness were registered. However, at the absorbed microwave power of 620 W, a significant increase of the stiffness can be seen. The results on Figure 7c show an irregular increase of the transition indent with the absorbed microwave power, while in the case of Figure 7d, generally speaking, a transition force decrease with each increase in the absorbed microwave power can be noted. Further non-monotonic changes of the mechanical properties of the plasma-treated PC surface, such as the deformation and the adhesion force, can be seen in Figure 7e,f, respectively. Although for each absorbed microwave power level the deformation parameter is higher than for the pristine sample, the adhesion force at the first stage grows and then decreases with an increase of absorbed microwave power. Based on the results presented in Figure 7 concerning the dependencies of the polycarbonate surface mechanical properties parameters on the microwave power, one can note that the obtained results confirm the complex and non-monotonic changes of the mechanical properties of the surface, which mostly cannot be interpreted simply. It can be related to the co-existing various mechanism induced by the plasma beam, appearing with various intensities at different plasma power values.

As it is known, the plasma treatment of the polymer’s surface changes not only the wettability, morphological, and mechanical properties of the sample surface, but modifications in the chemical binding structure are also observed. Since we did not measure changes in the surface chemistry induced by the plasma treatment, some valuable information can be found in the literature. Mostly, these data come from an X-ray photoelectron spectroscopy (XPS) technique, which provides spatial elemental analysis of the topmost atomic layers of polymeric materials surfaces. In that way, by measuring and comparing the XPS results on chemical composition of the PC sample surface before and after plasma treatments, the information about changes induced by the plasma can be obtained. 

As can be found in [65,67,68,69] the data results of the XPS of the pristine PC sample reveals C1s and O1s narrow spectra. According to Reference [67], the C1s spectra is characterized by five Gaussian functions corresponding to the five different bonding states of the carbon. These are as follows: Aromatic C-H bond (with binding energy of 284.5 eV); aliphatic C-H, C-C bond (with binding energy of 285 eV); aromatic C-O bond (with binding energy of 286.24 eV); C=O bond (with binding energy of 290.44 eV); and C shake up satellite (with binding energy of 291.67 eV). The O1s spectra is represented by carboxylic oxygen O=C (with binding energy of 532.33 eV) and singly bonded oxygen C-O-C (with binding energy of 533.97 eV) [67]. In comparison, in Reference [68] the following peaks of the C1s narrow spectra of the untreated PC sample were recognized: C-C, C-H (with binding energy of 285 eV); C-O (with binding energy of 286.5 eV); C=O, O-C=O (with binding energy of 288.4 eV); O-C(=O)–O (with binding energy of 290.5–291 eV); C shake up satellite (292.6). The following two components of the O1s narrow spectra were reported: Carboxylic oxygen O=C (with binding energy of 532.4 eV) and singly bonded oxygen C-O-C (with binding energy of 534 eV).

As it was reported in Reference [67] after argon plasma treatment of the PC sample surface, the significant decrease of the carboxylic carbon bonds and the C shake up satellite was noted, while the peaks of the aromatic C-H bond, aliphatic C-H, C-C bond, and aromatic C-O bond did not change significantly [65]. At the same time, the strong decrease of singly bonded oxygen C-O-C peak was observed. Similarly, according to Reference [65], the argon plasma treatment results in reduction of the carboxylic carbon bonds and the disappearance of the C shake up satellite. However, the percentage of the aliphatic C-H, C-C bond peak decreased and the percentage of aromatic C-O bond peak increased after plasma treatment. Additionally, contrary to Reference [67], the increase in carboxylic oxygen O=C bonds was noticed. Additionally, after argon plasma treatment of the PC sample surface, the decrease in the atomic concentration of carbon and increase in the atomic concentration of oxygen was observed. This increase of oxygen-to-carbon ratio confirms the increase of oxygen containing polar functional groups on the PC sample surface [65,70]. The increase in the oxygen-to-carbon ratio was also reported in the case of atmospheric pressure helium plasma treatment of PC material surface [35], however, as it was found out, it is a plasma treatment time dependent effect. The creation of the oxygen-containing polar functional groups on the PC material surface results in the increase in surface free energy, making at the same time the surface hydrophilic which explains the decrease in the contact angle after plasma treatment, also observed in our study.

It is also worth of mentioning here that instead of rare gases plasma (like argon plasma in our case), molecular gases plasma has been used for different polymeric material surface modification, e.g., oxygen plasma. In Reference [27], a variety of reactive gaseous species of oxygen plasma suitable for polymers surface modification are listed. Apart from free electrons, these are the following: neutral oxygen molecules in the metastable states; neutral oxygen atoms in the ground state; neutral oxygen atoms in excited states; positively charged oxygen molecules; negatively charged atomic ions; and negatively charged molecular ions. Due to this various functional groups as a result of oxygen plasma treatment, polymeric materials on the surface can be formed. These can be groups like C-O(H) (hydroxyl or ether), C-O-O (peroxy), C=O (carbonyl group in aldehydes or ketones), O=C-O(H) (carboxyl or ester) or OC(O)O (carbonate). Direct comparison of argon versus oxygen plasma treatment of the PC surface can be found in References [34,69,70]. As it was reported in [70], in the case of argon plasma treatment, no new functional groups are formed on the PC surface, while in the case of oxygen plasma treatment, various functional groups like C-O, C=O, O-C=O, and C-O-O are formed. During surface modification by an argon plasma, the roles are played by the direct (the ion bombardment of the surface) and radiative energy transfer processes (UV radiation emitted by the plasma). The surface modification by the oxygen plasma treatment is mainly caused by the reactions of atomic oxygen with the surface carbon atoms and the reactions between the oxygen plasma reactive species and the surface atoms. 

## 4. Conclusions

The presented results of our experimental studies contributed to the development of atmospheric pressure plasma-based technology for surface modification of polycarbonate materials. In contrast to the atmospheric pressure microwave plasma sources typically generating plasmas in the forms of flames or cylindrical columns, we utilised a new plasma source delivering plasma in the form of a regular sheet sustained within a flat dielectric box. It was operated at a standard microwave frequency of 2.45 GHz, at an absorbed microwave power up to 850 W, and at an argon flow rate up to 20 L/min. The roles analysed include the absorbed microwave power and the number of scans on the wettability and morphological and mechanical properties of the plasma-treated PC samples. The wettability of the plasma-treated PC samples was described by the water contact angle; the morphological properties were characterised by the average roughness (Sa), root mean square roughness (Sq), skewness (Ssk), kurtosis (Sku), peak-peak value (Sz) and surface area ratio (Sdr); and the mechanical properties were described by such parameters as Young’s modulus, stiffness, transition indent, transition force, deformation, and adhesion force. In order to investigate the changes of the above-mentioned parameters, water contact angle goniometry and atomic force microscopy were performed. The results of the investigations show that the microwave argon plasma sheet has a high potential for PC material surface modification. For example, one of the promising outcomes of the experiments is the reduction of the water contact angle from about 76° for a pristine PC sample to about 32° for a PC sample treated with the plasma one time at a microwave power level of 380 W.

The new type of atmospheric pressure microwave plasma presented can be used for surface modification of any type of plastic material, such as polyethylene (PE), polytetrafluoroethylene (PTFE), and polystyrene (PS), and can be scaled up relatively easily for industrial production lines for high-speed continuous processes of different material surface modifications.

## Figures and Tables

**Table 1:** Comparison of the water contact angle for different plasma-based methods of surface modification of polymeric materials.

Plasma Type	Plasma Forming Gas	Pressure	Sample Material	Water Contact Angle of Pristine Sample	Water Contact Angle After Plasma Treatment	Reference
DC plasma	Air	Low	Polytetrafluoroethylene	108.9°	74.2°	[20]
RF plasma	Air	Low	Polytetrafluoroethylene	108.9°	75.2°	[20]
Microwave plasma sheet	Argon	Atmospheric	Polyethylene	79.7°	29°	[25]
Glow discharge	Air	Low	Polypropylene	97.1°	32°	[30]
Glow discharge	Oxygen	Low	Polypropylene	97.1°	14°	[30]
DBD	Air	Atmospheric	Polycarbonate	81.5°	37.9°	[40]
Gliding arc	Air	Atmospheric	Polycarbonate	81.5°	30.4°	[40]
Corona discharge	Air	Atmospheric	Polycarbonate	80°	38°	[42]
Corona discharge	Air	Atmospheric	Cyclic olefin copolymer	96°	39°	[42]
Corona discharge	Air	Atmospheric	Cyclic olefinpolymer	93°	27°	[42]
Arc plasma	Argon	Atmospheric	Polyethylene	90°	38.5°	[66]
Microwave plasma sheet	Argon	Atmospheric	Polycarbonate	76°	32°	Present study
